# Molecular Identification and Biogenic Amine Production Capacity of *Enterococcus faecalis* Strains Isolated from Raw Milk

**DOI:** 10.3390/ijms262110480

**Published:** 2025-10-28

**Authors:** Patryk Wiśniewski, Federica Barbieri

**Affiliations:** 1 Department of Food Microbiology, Meat Technology and Chemistry, Faculty of Food Science, University of Warmia and Mazury, Plac Cieszyński 1, 10-726 Olsztyn, Poland; 2 Department of Agricultural and Food Sciences, University of Bologna, 47521 Cesena, Italy; federica.barbieri16@unibo.it

**Keywords:** dairy, tyramine, tyrosine decarboxylase

## Abstract

In this study, *Enterococcus faecalis* strains isolated from raw cow’s milk were examined for genetic diversity, ability to produce biogenic amines (including histamine, tyramine, putrescine, cadaverine, 2-phenylethylamine) and the presence of corresponding amino acid decarboxylase genes. Identification of 29 strains obtained from Polish farms was carried out by polymerase chain reaction (PCR) and matrix-assisted laser desorption and ionization time of flight (MALDI-TOF MS) methods, and their genetic relationships were assessed by the Enterobacterial Repetitive Intergenic Consensus Polymerase Chain Reaction (ERIC-PCR) technique. Amine production capacity was assessed in vitro on synthetic medium, while the presence of decarboxylase genes (*hdcA*, *tyrS*, *tyrDC*, *Odc*, *ldc*) was detected by molecular assays, with the use of optimized primers enabling the detection of *tyrDC* in strains previously considered negative. The results showed high variability between strains and the ability of some isolates to produce high concentrations of *tyrDC* (max. > 1000 mg/kg); the presence of the *tyrDC* gene was strongly correlated with high production, although *tyrDC*-positive strains with low production were also reported, suggesting the influence of regulatory or environmental factors. The study underscores the need for precise molecular tools and systematic monitoring of biogenic amines to ensure the safety and quality of dairy products.

## 1. Introduction

Ensuring the product safety at a microbiological level is a priority challenge for the food industry, to avoid consumer risks [1]. Food-safety management systems therefore rely on preventive practices—strict sanitation, control of cross-contamination and of processing parameters (temperature, pH, water activity), and careful selection of starter/adjunct cultures—to minimize contamination and the accumulation of harmful compounds. Among these, proper sanitization with strict standards of cleanliness, attention to cross-contamination, and the control of process parameters (e.g., temperature, pH) are needed [2]. These measures are intended to prevent the spread of foodborne pathogens and the accumulation of harmful microbial metabolites [3].

One group of these undesirable compounds, as an indicator of food spoilage [4], is represented by biogenic amines (BAs), which are responsible for adverse effects and are involved in several pathogenic syndromes in consumer health, depending on their concentration [5]. They are derived from bacterial decarboxylation of the corresponding amino acids through decarboxylase enzymes [6,7]. BAs are low-molecular-weight nitrogen compounds formed mainly as a result of bacterial decarboxylation of amino acids using specific decarboxylases. Typical BAs found in food are histamine and tyramine (heterocyclic and aromatic, respectively) and aliphatic amines such as putrescine and cadaverine. Polyamines (spermidine, spermine) have distinct biosynthesis pathways and functions and are often discussed separately, although putrescine is sometimes classified in both groups [8,9,10]. The most dangerous BAs found in foods are represented by histamine and tyramine due to the symptoms that they may cause (“fish poisoning” and “cheese reaction” syndrome). Their toxicity also depends on individual sensitivity or allergy, and the simultaneous monoamine oxidase inhibitory drugs or ethanol intake [11]. High levels of BAs in foods can lead to symptoms like hypertension, headaches, and allergic reactions. Recent studies have highlighted the toxicological effects of BAs and their synergistic potential, which could intensify adverse health outcomes [12].

Tyramine is the most common BA associated with enterococci, particularly in dairy and meat products, where it accumulates due to tyrosine decarboxylase activity [13]. Putrescine is another significant BA found in fermented foods, such as cheeses and soybean-based products, primarily synthesized via the ornithine decarboxylase or agmatine deiminase pathways [14]. Histamine and cadaverine, though less frequently linked to enterococci than other lactic acid bacteria, have been detected in specific fermentation processes [15]. 2-phenylethylamine has also been identified in several strains of enterococci used in artisanal cheese production [16].

The formation and level of BA in food depend on the development of microflora and factors such as storage time and temperature, food properties (water activity, pH), availability of free amino acids, and the presence of microorganisms with decarboxylase genes [4,17]. The decarboxylase enzyme activity is a characteristic of many groups of bacteria, including Enterobacteriaceae, Lactobacillaceae, *Clostridium* sp., *Streptococcus* sp., *Pseudomonas* sp. and *Enterococcus* sp. [7,18], leading to the formation of BAs in several food products.

Due to their ability to survive in harsh conditions, enterococci are a widespread microbiota in many products. Their presence in foods is controversial: on one hand, some enterococci are considered probiotics, with health benefits, while on the other hand, they are characterized by a high frequency of antibiotic resistance, the presence of many virulence factors, or the production of BAs [19,20,21,22]. Several species of *Enterococcus* (especially *Enterococcus faecalis*, *Enterococcus faecium*, *Enterococcus durans*, and *Enterococcus mundtii*) are well-documented producers of tyramine, accumulating it during fermentation through tyrosine decarboxylase activity. They may also contribute to putrescine formation through ornithine decarboxylase (ODC) and agmatine deiminase (ADI, *aguBDAC*) pathways. Ladero et al. [18] suggested that the capability to produce tyramine can be considered a species-level trait in *E. faecalis*, but is also widespread in *E. faecium*, *E. durans* and *E. mundtii*. Moreover, it is reported that they can produce other biogenic amines, including putrescine [5,22]. The biosynthesis of putrescine in *E. faecalis* occurs mainly through two distinct metabolic pathways: the ornithine decarboxylase (ODC) pathway and the agmatine deiminase (ADI) pathway. While the ODC pathway converts ornithine into putrescine via ornithine decarboxylase (encoded by the *odc* gene), the ADI pathway transforms agmatine into putrescine through a set of enzymes encoded by the *aguBDAC* operon [23,24]. Regulation involves response regulators such as *aguR*, as well as co-regulation between agmatine/tyramine clusters [25]. Although enterococci contribute to BA formation in dried fermented meat products and some cheeses, their frequent occurrence of antibiotic resistance determinants and virulence factors makes them problematic as starter or probiotic strains [26]. Therefore, modern strain selection criteria explicitly include the inability to produce BAs (absence of decarboxylase genes and no measurable formation of amines under appropriate conditions) in addition to the absence of transferable resistance and virulence traits.

Therefore, this study aimed to investigate the ability of *Enterococcus faecalis* strains isolated from cow raw milk to produce selected biogenic amines (histamine, tyramine, putrescine, cadaverine, and 2-phenylethylamine), and to determine the presence of genetic determinants encoding amino acid decarboxylase enzymes responsible for BA production. To improve the accuracy of gene detection, different sets of primers targeting the same decarboxylase genes were used, particularly for *tyrDC*, to assess their effectiveness and highlight the relevance of proper primer selection in molecular screening.

## 2. Results and Discussion

Enterococci are recognized as an important component of the natural microbiota of milk and dairy products [27,28]. They may include strains capable of producing biogenic amines. In many traditional fermented products such strains occur naturally and contribute to the fermentation process rather than representing classical spoilage microorganisms. Most enterococcal strains reported in the literature are tyramine producers [29]. While several authors have reported correlation between the number of enterococci and the concentration of tyramine [5,25] and putrescine [5,18,25] in dairy products, the presence of biogenic-amine-producing strains does not by itself imply a health hazard—the potential risk depends on the amount of biogenic amines produced and whether their concentration exceeds thresholds that are significant from a toxicological point of view. For this reason, accurate molecular characterization of strains and systematic monitoring of biogenic amine levels are necessary to assess both quality and safety.

Although biogenic amines can cause negative effects in sensitive individuals, raw milk that undergoes routine handling and transportation does not usually promote bacterial growth to a degree sufficient to produce high concentrations of amines. The main risk is associated with further processing (e.g., traditional fermentation) or prolonged improper storage, which may allow the proliferation of bacteria that produce amines. Therefore, risk assessments should be based on quantitative data on amines and contextual information on the use and storage of milk. Mainly responsible for it are 3 species of enterococci: *E. faecium*, *Enterococcus durans* and *E. faecalis* [18]. Studies on the ability of *E. faecalis* isolated from raw milk to produce biogenic amines are an important step in assessing the potential health risk posed by the presence of this microorganism in dairy products.

### 2.1. Genotypic Similarity of E. faecalis Strains Based on ERIC-PCR Analysis

**Figure 1:**
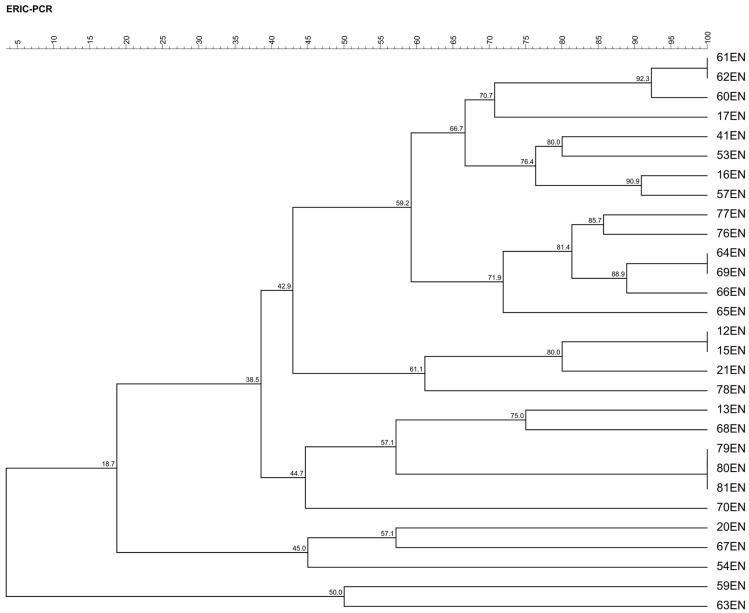
The UPGMA dendrogram obtained from ERIC-PCR profiles shows similarity in the restriction pattern.

The dendrogram obtained from ERIC-PCR analysis, constructed using the UPGMA (unweighted pair group method with arithmetic mean) algorithm, shows the hierarchical structure of genetic similarity between the *E. faecalis* strains studied (Figure 1). Similarity levels ranged from 18.7% to 100%, indicating the presence of both closely related and genetically distinct strains. The highest similarity (100%) was observed between strain pairs 12EN and 15EN, 61EN and 62EN, 64EN and 69EN, and 79EN, 80EN and 81EN, suggesting potential clonal origin or very recent common ancestry. At a similarity threshold of approximately 60%, the dendrogram reveals the presence of distinct genetic clusters, highlighting potential differences in evolutionary divergence among the strains. Notably, no clear relationship was observed between clustering patterns and the geographic origin of the isolates (Warmian–Masurian vs. Kuyavian–Pomeranian Voivodeships), suggesting that genetic variability may be influenced by other factors such as environmental conditions, farm-level practices, or selective pressure.

This observed genetic diversity prompts further investigation into the functional implications of these variations. Genetic analysis of *E. faecalis* isolated from raw milk can provide valuable insights into potential virulence determinants and the presence of genes responsible for biogenic amine synthesis [30].

### 2.2. Production of Biogenic Amines Among Enterococcus faecalis Strains

The potential production of BAs was tested for each selected *E. faecalis* strain in a synthetic microbial medium, added with specific amino acid precursors. Histidine, tyrosine, 2-phenylalanine, ornithine, and lysine were added to observe the accumulation of histamine, tyramine, 2-phenylethylamine, putrescine, and cadaverine, respectively. The HPLC results for the 29 *E. faecalis* strains (Table 1) showed a widespread ability to produce tyramine and 2-phenylethylamine among the samples, while histamine, putrescine, and cadaverine were under the detection limit (3 mg/L) in all of them. Significant differences were observed in the levels of biogenic amines between different bacterial strains of the same species. Tyramine production characterized most of the tested strains (82.8%) with levels ranging from 791.96 ± 94.81 mg/L to 1081.27 ± 84.97 mg/L. The highest concentrations of tyramine are found in strains 76EN (1081.27 ± 84.97 mg/L), 60EN (1080.08 ± 103.60 mg/L) and 20EN (1057.76 ± 112.50 mg/L), indicating a strong presence of tyrosine decarboxylase activity within these strains. On the other hand, the 17.2% tested strains (54EN, 63EN, 68EN, 70EN and 81EN) show minimal tyramine levels (below 15 mg/L), suggesting variability in the enzyme activity or gene expression among these samples. *E. faecalis,* able to produce high concentrations of tyramine, can pose a health risk to consumers. Consumption of products containing high levels of tyramine can lead to hypertensive reactions in susceptible individuals, which is especially true for dairy products. The literature emphasizes that the toxic effects of individual biogenic amines are evident even at relatively low concentrations [31]. According to Bover-Cid et al. [32], histamine can cause adverse effects at different doses, depending on individual sensitivity. The toxic threshold levels for histamine range from 8–40 mg (mild poisoning) to 1500–4000 mg (severe poisoning), with histamine-containing fish poisoning (HFP) often associated with levels between 600 and 3000 mg/kg in fish products [32]. According to Regulation (EC) 2073/2005 [33], the maximum permissible levels of histamine in fresh fish are 100–200 mg/kg and in salted fish products up to 400 mg/kg [33]. In the case of tyramine, hypertensive reactions have been reported after consumption of 200–2000 mg in healthy individuals, while migraine sufferers may react to doses as low as 100 mg [32]. In addition, according to recent studies, newer generation monoamine oxidase inhibitors (MAOIs) (RIMAs) tolerate up to 50–100 mg, while in the average population, a dose equivalent to 200–800 mg of tyramine causes hypertensive crises [34]. The proposed upper limit for the sum of biogenic amines (histamine, tyramine, putrescine, cadaverine, etc.) in food is 750–900 mg/kg [35]. However, even in strains carrying this gene, the level of tyramine production can vary due to specific regulatory factors and the availability of precursors in the culture medium. The literature repeatedly describes the need to monitor levels of biogenic amines in food products, especially meat and dairy, to reduce the health risks associated with their consumption [18]. Research has shown that enterococci strains exhibit varying tyramine production capacities based on growth conditions [36]. For example, pre-culture in tyrosine-enriched environments enhances decarboxylase activity. Factors such as pH between 5 and 6.5 and high moisture content in the medium are optimal for tyramine production. Conversely, lack of precursors or unfavorable environmental conditions can lead to diminished or no tyramine production despite the presence of the gene [36]. Further studies, including analysis of regulatory sequences and gene expression, could help explain the mechanisms responsible for such a large variation in tyramine production in strains with apparently similar genetic profiles.

2-phenylethylamine is another BA that the analyzed enterococci produced at concentrations ranging from 104.94 ± 11.55 mg/L to 186.58 ± 26.40 mg/L, though its levels are much lower compared to tyramine. The highest concentrations are found in strains 76EN (186.58 ± 26.40 mg/L) and 77EN (185.44 ± 26.02 mg/L), followed by 60EN (177.06 ± 32.90 mg/L) and 20EN (178.54 ± 37.56 mg/L). It is important to note that the same strains that were not characterized by a tyramine production were also unable to accumulate a huge amount of 2-phenylethylamine. This observation is consistent with recent findings indicating that both tyramine and 2-phenylethylamine can be synthesized by the same enzyme, tyrosine decarboxylase (TyrDC). A knockout study conducted by Pérez et al. [37] demonstrated that deletion of the *tdcA* gene in *E. durans* abolished the production of both tyramine and 2-phenylethylamine, providing direct evidence that TyrDC catalyzes the decarboxylation of phenylalanine as well. Although the presence of the *tyrDC* gene is a prerequisite for this biosynthetic capability, its expression and enzymatic activity are known to be modulated by strain-specific regulatory mechanisms and environmental conditions [38,39]. Consequently, even *tyrDC*-positive strains may vary in their actual production levels. The literature suggests that 2-phenylethylamine production is less common among lactic acid bacterial strains and usually occurs at lower levels than tyramine [5]. Moreover, genotypic and phenotypic characteristics of these bacteria may vary depending on environmental conditions, which can significantly influence their biochemical activity and potential toxicity [38,40]. For instance, the microbial composition and their activity in raw milk or artisanal cheeses can be influenced by environmental conditions, such as temperature, humidity, and seasonal changes in animal feed [41]. These factors can modulate the ability of *Enterococcus* species to produce biogenic amines, as well as their antimicrobial resistance profiles [42].

Although no accumulation of putrescine was detected in any of the analyzed *E. faecalis* strains under the tested conditions, this absence must be interpreted with caution. The biosynthesis of putrescine in *E. faecalis* primarily occurs via the agmatine deiminase pathway, which requires the presence of agmatine in the environment as a substrate [24]. In our study, the medium was supplemented with ornithine, thus favoring the detection of the ornithine decarboxylase pathway. Consequently, the lack of putrescine detection may reflect pathway-specific activation rather than a true inability of these strains to produce this compound. Previous research has identified the *aguBDAC* operon responsible for agmatine conversion to putrescine and confirmed its activity in various dairy and fermented food isolates [23,25]. Therefore, the true putrescine-producing potential of these strains remains to be determined.

The complete absence of histamine, cadaverine, and ornithine-derived putrescine in all strains indicates focus to tyramine and 2-phenylethylamine as the main biogenic amines, with potential implications for food safety given the high levels of tyramine observed in many strains. The data indicate that *E. faecalis* strains are potential sources of tyramine and 2-phenylethylamine, with the variability in production possibly influenced by genetic factors, environmental conditions, or strain-specific metabolic regulation. Future research on BAs in dairy products should focus on developing innovative control strategies and understanding the underlying mechanisms of BA production and degradation. One promising area involves the use of enzymatic reduction by lactic acid bacteria, such as *Lacticaseibacillus casei*, which has demonstrated the ability to degrade BAs like histamine and putrescine in dairy environments [43].

### 2.3. Presence of Genes Associated with the Accumulation of Biogenic Amines Among Selected Strains

In our study, none of the selected enterococcal strains tested positive for the presence of histidine decarboxylase (*hdcA*), lysine decarboxylase (*ldc*), or ornithine decarboxylase (*Odc*) genes using primers HdC1/HdC2, Cad2F/Cad2R, and ODF/ODR, respectively. Only genes involved in tyramine synthesis (*tyrDC* and *tyrS*) were detected in the tested strains, as shown in Table 2. According to the literature, the ability to produce tyramine and 2-phenylethylamine accumulation is observed among enterococci; however, these strains are not able to produce cadaverine and putrescine [44]. The results of our study are consistent with those of Inoğlu and Tuncer [45], in which none of the analyzed enterococci strains possessed the *hdc*, *ldc*, and *Odc* genes. It is important to note, however, that the main genetic cluster responsible for putrescine biosynthesis in *E. faecalis* is the agmatine deiminase pathway, which includes the *aguA*, *aguB*, *aguC*, *aguD*, and *aguR* genes. These genes encode enzymes and regulatory elements necessary for converting agmatine to putrescine and were not targeted in our current PCR screening [24].

In total, twenty-one strains (72.41%) carried both *tyrS* and *tyrDC.* The remaining eight strains (27.59%)—i.e., 41EN, 57EN, 59EN, 60EN, 66EN, 68EN, 78EN and 79EN—possessed only the *tyrDC* gene, while lacking *tyrS*. Among these eight *tyrS*-deficient strains, as many as seven had high tyramine production (>900 mg/L). The only exception was strain 68EN, whose tyramine production capacity was much lower than <20 mg/L. Additionally, five strains (54EN, 63EN, 68EN, 70EN, and 81EN) produced only trace amounts of tyramine (<10 mg/L) despite carrying the *tyrDC* gene, which indicates that the mere presence of this gene does not guarantee its expression or functional activity. Similarly, a study by Bargossi et al. [46] showed that although *tyrDC* gene was common in enterococci, strains differed significantly in decarboxylase activity. In other bacteria, the gene may be silent. Eom et al. [47] observed isolates with intact histidine and tyrosine decarboxylase genes, but no expression of these enzymes and no histamine or tyramine production, respectively. These findings underscore the complexity of the genetic and environmental regulation of biogenic amine biosynthesis. The observed variability in tyramine production among genetically similar strains suggests that additional regulatory mechanisms, including alternative transcription factors or strain-specific expression controls, may influence *tyrDC* activation [5,37,48]. Environmental factors are known to play a key role in regulating *tyrDC* gene expression and tyramine biosynthesis. Environmental and nutritional factors further modulate *tyrDC* expression and activity. For example, acidic conditions combined with abundant tyrosine strongly induce the pathway. Perez et al. [49] showed that low pH, together with ample substrate, triggers *tyrDC* transcription and boosts tyramine output. Strains incubated at elevated temperatures (e.g., 45 °C) produced substantially higher amounts of tyramine compared to those grown at lower temperatures, such as 25 °C [50]. Osmotic stress is another important modulatory factor. Liu et al. [51] demonstrated that NaCl stress led to upregulation of both *tyrDC* and the downstream *tyrP* gene encoding the tyrosine/tyramine antiporter in *E. faecalis*, resulting in increased tyramine accumulation under specific stress conditions. These findings indicate that parameters such as pH, temperature, salt concentration, and precursor (tyrosine) availability can dramatically alter the transcriptional activity of the *tyrDC* gene and the enzymatic function of tyrosine decarboxylase. As a result, these factors introduce additional layers of physiological control over tyramine formation in enterococci [47,49].

The locus influencing the ability to produce tyramine usually includes genes encoding tyrosine decarboxylase (*tyrDC*), tyrosyl tRNA synthetase (*tyrS*, located upstream of the *tyrDC* gene), putative tyrosine/tyramine permease (*tyrP*, located downstream of the *tyrDC* gene) and Na+/H+ antiporter (*nhaC*) [52]. The selection of this specific gene locus for analysis was based on its well-documented and central role in tyramine biosynthesis in enterococci. The *tyrDC* gene encodes the key enzyme responsible for tyramine production, while its adjacent genes (including the *tyrS* gene) form a conserved *tdc* cluster frequently co-transcribed and functionally associated with regulation, transport, and adaptation to environmental stress. Investigating this region allowed not only for confirmation of tyramine-producing potential but also for a deeper understanding of genetic determinants potentially affecting strain-specific variation in amine biosynthesis. The researchers indicate the organization of different *tdc* clusters, their distribution, and high sequence similarity, indicating a horizontal transfer of this cluster from a common source [53]. Moreover, horizontal gene transfer (HGT) has been shown to play a key role in the spread of genes responsible to produce biogenic amines—increasing the metabolic flexibility and adaptability of bacterial populations in cheese microbiomes—and this spread is often mediated by mobile genetic elements such as plasmids and integrons [54,55].

The results of this study indicate that the key element in tyramine biosynthesis in the enterococci strains studied is the *tyrDC* gene, regardless of the primer set used (DEC5/DEC3 or Tdc-F2/Tdc-R2; Table 3). Detection of the *tyrDC* gene using two different primer pairs (DEC5/DEC3 and Tdc-F2/Tdc-R2) indicates a high probability of the actual presence of this gene in the analyzed material. The use of two independent primer systems, each targeting distinct regions of the *tyrDC* gene, increases the overall reliability of detection and minimizes the risk of false-positive or false-negative results due to non-specific binding, sequence variability, or template degradation. Using both sets in parallel helps compensate for possible mismatches or partial DNA degradation that might prevent one primer pair from binding effectively—under such conditions, the alternate pair may still succeed. Concordant amplification by both primer sets, therefore, serves as confirmatory evidence for *tyrDC* presence, reducing the likelihood of spurious results or amplification dropouts. This approach allows for a more precise identification of microorganisms capable of tyramine production, a trait of toxicological relevance in the context of food safety. The presence of the *tyrDC* gene in all 29 strains analyzed strongly suggests that the enzyme tyrosine decarboxylase, encoded by this gene, plays a key role in the biosynthesis of this biogenic amine, which is consistent with previous studies [45,46,56,57,58]. Further studies, including analysis of regulatory sequences and gene expression, could help explain the mechanisms responsible for such a large variation in tyramine production in strains with apparently similar genetic profiles.

### 2.4. Limitations and Future Work

A key limitation of the present study is the focus on the ornithine decarboxylase (ODC) pathway to assess putrescine biosynthesis, without consideration of the agmatine deiminase pathway. As demonstrated by Landete et al. [24] the ADI pathway constitutes the primary route for putrescine formation in *E. faecalis*, driven by the *aguBDAC* gene cluster. Moreover, putrescine production via this route is inducible by the presence of agmatine in the growth medium. Future studies should therefore incorporate agmatine supplementation and PCR amplification of the *agu* operon to fully evaluate the putrescine-producing capacity of *E. faecalis* strains. This will provide a more complete understanding of their potential impact on biogenic amine accumulation in dairy products.

## 3. Materials and Methods

### 3.1. Selected Strains Used in This Study

The 29 *Enterococcus faecalis* strains used in this study belonged to the collection of the Department of Food Microbiology, Meat Technology and Chemistry at the University of Warmia and Mazury in Olsztyn (Poland). These strains were previously isolated from raw milk samples collected from various farms located in the Warmian–Masurian and Kuyavian–Pomeranian Voivodeships in Poland. These strains were previously isolated from raw bulk milk samples (100 mL each), collected aseptically during routine milking by instructed personnel into sterile containers. All samples were obtained in 2022 from clinically healthy cows. According to farmers’ declarations, no antibiotic treatments had been applied within 30 days prior to sampling. The cows were kept under pasture housing conditions. Table S1 (Supplementary Material) lists the farm identifiers, geographical areas, sampling dates, host species, health status, recent antibiotic use, housing, and sampling method. Different presumptive *Enterococcus* isolates were initially obtained using Slanetz–Bartley agar (Merck, Darmstadt, Germany), and species identification confirmed them as *E. faecalis* using two methods—PCR and MALDI-TOF MS (bioMérieux, Marcy-l’Étoile, Frnace). The isolates were stored at −80 °C in microbanks (Biomaxima, Lublin, Poland) and cultured on Tryptic Soy Agar (TSA; Merck, Darmstadt, Germany) at 37 °C in 24 h before further analyses.

#### 3.1.1. PCR Identification

All presumptive enterococci isolates were genotypically identified by PCR. Genomic DNA was purified using the Genomic Mini kit (A&A Biotechnology, Gdańsk, Poland), according to the manufacturer instructions. The obtained genomic DNA was stored at −20 °C for further analysis. Then, the identification at the species level was performed by PCR, using specific primers described by Zarzecka et al. [65] (Table 4). PCR reaction products were separated by electrophoresis in a 1.5% agarose gel (m/v), stained with 0.5 μg/mL ethidium bromide (Sigma-Aldrich, St. Louis, MO, USA), and visualized under UV light using the G-BOX gel analysis and documentation system (Syngene, Cambridge, UK).

#### 3.1.2. MALDI-TOF MS Identification

All isolates were also identified using MALDI-TOF MS (matrix-assisted laser desorption and ionization time of flight). Measurements were performed using the VITEK^®^ MS system (Applied Maths/bioMérieux, Sint-Martens-Latem, Belgium) according to the methodology described by Wiśniewski et al. [66] and Zakrzewski et al. [67] with an acceleration voltage of 200 kV, a mass range of 2–20 kDa, a laser frequency of 50 Hz, and an extraction delay time of 200 ns. Mass fingerprints were analyzed via the VITEK^®^ MS v2.0 MALDI-TOF system (RUO; SARAMIS v4.13).

Isolates were tested in duplicate using the direct transfer protocol. After 48 h of incubation at 30 °C in TSA (Merck, Darmstadt, Germany), they were transferred to the target plate, treated with 1 µL of MALDI matrix VitekMS-CHCA, and analyzed. Confidence levels were expressed as percentages.

### 3.2. Genotyping of Strains Using ERIC-PCR

The strains were genotyped using ERIC-PCR (Enterobacterial Repetitive Intergenic Consensus Polymerase Chain Reaction) following the protocol outlined by Zarzecka et al. [68] and Wiśniewski et al. [69]. The reaction was conducted using a Mastercycler Nexus GX2 Thermocycler (Eppendorf, Hamburg, Germany) in a 25 μL reaction volume, including 12.5 μL of DreamTaq Green PCR Master Mix (2×) (ThermoFisher Scientific, Waltham, MA, USA), 1.25 μL of both 10 pmol primers ERIC1 (5′-ATG TAA GCT CCT GGG GAT TCA-3′) and ERIC2 (5′-AAG TAA GTG ACT GGG GTG AGC G-3′) (Genomed, Gdańsk, Poland), 1.5 μL of DNA template (50 ng/μL), and nuclease-free water to reach the final volume. The PCR reaction conditions included an initial denaturation at 95 °C for 7 min to effectively separate DNA strands, followed by 30 cycles consisting of denaturation at 94 °C for 1 min, primer attachment at 52 °C for 1 min, and elongation at 65 °C for 8 min. Finally, a final elongation at 65 °C for 15 min was performed to ensure that all PCR products were fully synthesized.

The PCR products were separated using electrophoresis on a 1.5% agarose gel prepared with TBE buffer (1% Tris-borate-EDTA) and stained with 0.5 μg/mL ethidium bromide (Sigma-Aldrich, St. Louis, MO, USA). The bands were visualized using the G-BOX F3 system (Syngene International Limited, Bengaluru, KA, India). Cluster analysis was conducted using BioNumerics version 7.6.3 (Applied Maths/bioMérieux), employing the Dice coefficient for similarity calculations and generating dendrograms with the unweighted pair group method with arithmetic mean (UPGMA).

### 3.3. Screening of Biogenic Amine Production

Determination of the potential of enterococci strains to produce biogenic amines (histamine, tyramine, putrescine, cadaverine, and 2-phenylethylamine) was carried out by a screening method using the medium proposed by Bover-Cid and Holzapfel [70]. Overnight cultures incubated at 30 °C in Brain Hearth Infusion (BHI) broth (Merck, Darmstadt, Germany) were used at a concentration of 6 log CFU/mL in Bover-Cid and Holzapfel broth, added with the specific BA precursors (Table 5) and incubated at 37 °C, under aerobic conditions. This step was performed three times. Biogenic amine production was confirmed by high-performance liquid chromatography (HPLC) analysis, according to the method reported by Barbieri et al. [71]. Briefly, the obtained samples were centrifuged at 6000 rpm for 10 min, and the resulting cell-free supernatants were analyzed using an Agilent Instrument 1260 Infinity HPLC instrument (Agilent technologies, Inc., Santa Clara, CA, USA) with an automatic injector (G1329B ALS 1260, 20 μL loop) equipped with a UV detector (G1314F VWD 1260) set at 254 nm. Before injection, samples were derivatized with dansyl chloride (Sigma Aldrich, St. Louis, MO, USA) according to the method of Martuscelli et al. [72]. A C18 Waters Spherisorb ODS-2 column (150 × 4.6 mm, 3 μm) was used for chromatographic separation. The amounts of amines were reported in mg/L, based on a calibration curve obtained using aqueous dansyl-chloride-derivatized amine standards (Sigma-Aldrich, St. Luise, MO, USA). The detection limit of the analysis under the applied conditions was 3 mg/L. All samples were analyzed in triplicate.

### 3.4. Detection of the Genes Related to the Production of Biogenic Amines

The PCR method was used to determine the presence of genes related to the potential production of biogenic amines, such as *hdcA*, *tyrS*, and *tyrDC*, *Odc*, and *ldc* encoding histidine, tyrosine, ornithine, and lysine decarboxylase, respectively. The primers used in this study are listed in Table 3. The amplification reaction was performed in a thermocycler (Mastercycler nexus GX2/GX2e; Eppendorf, Hamburg, Germany). A 25 µL reaction mixture was used, which contained 2 µL of template DNA (50 ng/µL), 1X Easy-Taq buffer, 10 mM of dNTPs (deoxyribonucleotides), 0.4 µM of each primer, and 1.5 U Easy-Taq enzyme. The rest of the volume was made up of nuclease-free water. Amplification reactions were performed according to the parameters shown in Table 6. Each PCR cycle included positive and negative controls. Genomic DNA from *E. faecalis* ATCC 29212 was used as a positive control for the *tyrDC* gene, while genomic DNA from bacterial strains belonging to the strain collection in which the other genes analyzed in our study were identified was used as positive controls for the respective targets. Negative controls (nuclease-free water) were included in each run to monitor for contamination. PCR products were subjected to an electrophoresis analysis on a 1.5% agarose gel. Then the image of the resulting bands was visualized under UV light after ethidium bromide staining using the G-BOX F3 fluorescent stained gel documentation and analysis system (Syngene International Limited, Bengaluru, KA, India).

## 4. Conclusions

*Enterococcus faecalis* strains exhibit strain-specific biogenic amine production, with tyramine synthesis strongly associated with the presence of the *tyrDC* gene. However, because biogenic amine production depends on multiple factors, including regulatory mechanisms and environmental conditions, culture-based detection methods may yield false-negative results. Therefore, the presence of biosynthetic genes such as *tyrDC* is considered the most reliable indicator of a strain’s potential to accumulate biogenic amines in food products. In vitro assays revealed significantly higher tyramine levels compared to 2-phenylethylamine, reflecting the enzyme’s greater affinity for tyrosine. The application of two complementary primer sets (DEC5/DEC3 and Tdc-F2/Tdc-R2) enhanced detection reliability across genetically diverse strains, minimizing false negatives. While our controlled conditions clarify the strains’ intrinsic amine-producing potential, actual accumulation in dairy matrices may vary with pH, temperature, microbial interactions, and substrate availability, underscoring the need for in-product validation. Although putrescine was not detected in this study, the possibility of its biosynthesis via the agmatine deiminase pathway remains open. Further investigations involving agmatine supplementation and detection of *agu* genes are needed to fully characterize putrescine production potential. These findings support the implementation of routine molecular screening and targeted process controls to mitigate biogenic amine risks in fermented foods.

## Figures and Tables

**Table 1:** Detection of tyramine and 2-phenylethylamine accumulation in *E. faecalis* strains. Data are reported in mg/L as a mean of three repetitions.

No.	Strain	Biogenic Amine (mg/L)	
		Tyramine	2-Phenylethylamine
1.	12EN	875.98 ± 87.56	109.09 ± 21.01
2.	13EN	808.84 ± 101.20	154.14 ± 14.23
3.	15EN	791.96 ± 94.81	150.18 ± 36.11
4.	16EN	815.19 ± 97.44	158.87 ± 24.36
5.	17EN	820.55 ± 80.03	153.96 ± 10.05
6.	20EN	1057.76 ± 112.50	178.54 ± 37.56
7.	21EN	909.52 ± 101.66	155.09 ± 22.80
8.	41EN	999.76 ± 102.60	169.36 ± 16.00
9.	53EN	984.29 ± 88.65	167.92 ± 13.80
10.	54EN	7.10 ± 3.11	6.68 ± 1.57
11.	57EN	918.20 ± 76.36	150.79 ± 25.98
12.	59EN	968.86 ± 90.88	104.94 ± 11.55
13.	60EN	1080.08 ± 103.60	177.06 ± 32.90
14.	61EN	973.37 ± 94.00	176.55 ± 17.45
15.	62EN	983.67 ± 106.70	175.01 ± 20.08
16.	63EN	10.96 ± 3.05	7.99 ± 2.54
17.	64EN	1043.06 ± 114.03	175.74 ± 40.60
18.	65EN	948.52 ± 94.33	163.74 ± 18.54
19.	66EN	984.26 ± 70.96	177.41 ± 20.60
20.	67EN	933.08 ± 85.69	141.18 ± 35.40
21.	68EN	12.00 ± 4.90	6.97 ± 1.15
22.	69EN	1022.67 ± 83.47	176.11 ± 30.87
23.	70EN	7.73 ± 1.59	7.36 ± 2.24
24.	76EN	1081.27 ± 84.97	186.58 ± 26.40
25.	77EN	1037.58 ± 104.51	185.44 ± 26.02
26.	78EN	1055.50 ± 100.60	166.72 ± 13.55
27.	79EN	942.73 ± 86.50	166.37 ± 21.41
28.	80EN	1034.40 ± 99.02	171.62 ± 25.10
29.	81EN	11.21 ± 2.50	8.37 ± 3.01

**Table 2:** Presence of tyramine-associated genes (*tyrS*, *tyrDC*) among *Enterococcus faecalis* strains.

No.	Strain	*tyrS*	*tyrDC*
1.	12EN	+	+
2.	13EN	+	+
3.	15EN	+	+
4.	16EN	+	+
5.	17EN	+	+
6.	20EN	+	+
7.	21EN	+	+
8.	41EN	−	+
9.	53EN	+	+
10.	54EN	+	+
11.	57EN	−	+
12.	59EN	−	+
13.	60EN	−	+
14.	61EN	+	+
15.	62EN	+	+
16.	63EN	+	+
17.	64EN	+	+
18.	65EN	+	+
19.	66EN	−	+
20.	67EN	+	+
21.	68EN	−	+
22.	69EN	+	+
23.	70EN	+	+
24.	76EN	+	+
25.	77EN	+	+
26.	78EN	−	+
27.	79EN	−	+
28.	80EN	+	+
29.	81EN	+	+

+: detected; −: not detected.

**Table 3:** The primer sequences used to identify genes encoding decarboxylase enzyme to produce biogenic amines.

Enzyme	Gene	Primer Name	Sequence 5′-3′	Expected Amplicon Size (bp)	Reference
Histidine decarboxylase	*hdcA*	HdC1	TTGACCGTATCTCAGTGAGTCCAT	174	[59]
		HdC2	ACGGTCATACGAAACAATACCATC		
Tyrosine decarboxylase	*tyrS*	TD2	ACATAGTCAACCATGTTGAA	1100	[60]
		TD5	CAAATGGAAGAAGAAGTAGG		
	*tyrDC*	DEC5	CGT TGT TGG TGT TGT TGG CAC NACNGA RGA RG	350	[61]
		DEC3	CCG CCA GCA GAA TAT GGA AYR TAN CCC AT		
		Tdc-F2	CAA ATG GAA GAA GAA GT(A/T) GGA	1340	[62]
		Tdc-R2	CC(A/G/T) GCA CG(G/T) T(C/T)C CAT TCT TC		
Ornithine decarboxylase	*Odc*	ODF	CATCAAGGTGGACAATATTTCCG	500	[63]
		ODR	CCGTTCAACAACTTGTTTGGCA		
Lysine decarboxylase	*ldc*	Cad2F	CAYRTNCCNGGNCAYAA	1185	[64]

**Table 4:** The primer sequences used to identify the presumed enterococci [65].

Species	Primer Name	Primer Sequence 5′-3′	Product Size (bp)	Attachment Temperature (°C)
*Enterococcus* sp.	Ent-F	TCAACCGGGGAGGGT	733	60
	Ent-R	ATTACTAGCGATTCCGG		
*E. faecalis*	Fas-F	TCAAGTACAGTTAGTCTTTATTAG	941	54
	Fas-R	ACGATTCAAAGCTAACTGAATCAGT		
*E. faecium*	Fam-F	TTGAGGCAGACCAGATTGACG	658	54
	Fam-R	TATGACAGCGACTCCGATTCC		
*E. casseliflavus*	Cas-F	CGGGGAAGATGGCAGTAT	488	54
	Cas-R	CGCAGGGACGGTGATTTT		
*E. gallinarum*	Gal-F	GGTATCAAGGAAACCTC	822	54
	Gal-R	CTTCCGCCATCATAGCT		
*E. hirae*	Hi-R	TTTTGTTAGACCTCTTCCGGA	377	55
	Hi-F	TGAATCATATTGGTATGCAGTCCG		

**Table 5:** Summary of tested amino acid precursors for the formation of specific biogenic amines.

Amino Acid	Biogenic Amine
Histidine	Histamine
Tyrosine	Tyramine
Ornithine	Putrescine
Lysine	Cadaverine
Phenylalanine	2-phenylethylamine

**Table 6:** Specific amplification reaction parameters for each selected gene.

Enzyme	Gene	Primer Pair	Protocol	n° Cycles	Reference
Histidine decarboxylase	*hdcA*	HdC1/HdC2	Initial denaturation—94 °C, 2 min Denaturation—94 °C, 30 s Annealing—52 °C, 40 s Extension—72 °C, 30 s	35	[73]
Tyrosine decarboxylase	*tyrS*	TD2/TD5	Initial denaturation—95 °C, 5 min Denaturation—95 °C, 45 s Annealing—52 °C, 30 s Extension—72 °C, 1 min	31	
	*tyrDC*	DEC5/DEC3	Initial denaturation—94 °C, 5 min Denaturation—94 °C, 30 s ^1^; 90 °C, 30 s ^2^ Annealing—47 °C, 90 s ^1^; 50 °C, 1 min ^2^ Extension—72 °C, 1.5 min ^1^; 72 °C, 1 min ^2^ Final extension—72 °C, 7 min	5 ^1^; 30 ^2^	[61]
		Tdc-F2/Tdc-R2			
Ornithine decarboxylase	*Odc*	ODF/ODR	Initial denaturation—95 °C, 5 min Denaturation—95 °C, 45 s Annealing—52 °C, 30 s Extension—72 °C, 1 min	31	[73]
Lysine decarboxylase	*ldc*	Cad2F/Cad2R	Initial denaturation—94 °C, 2 min Denaturation—94 °C, 30 s Annealing—52 °C, 30 s Extension—72 °C, 1 min 30 s	30	

Abbreviations: n°—Number of cycles; ^1^ and ^2^ refer to the time applied for the respective number of amplification cycles.

## Data Availability

The data presented in this study are available in this article and Supplementary Materials.

## Supplementary Materials

The following supporting information can be downloaded at: https://www.mdpi.com/article/10.3390/ijms262110480/s1.
