# Supplementary material for: Molecular Identification and Biogenic Amine Production Capacity of Enterococcus faecalis Strains Isolated from Raw Milk
Source: Int J Mol Sci. 2025 Oct 28;26(21):10480. doi: 10.3390/ijms262110480 (PMC12608866; doi:10.3390/ijms262110480)
Supplement: Supplementary file 1 [file ijms-26-10480-s001.zip › ijms-3881186-supplementary.pdf]

Table S1. List of analysed *Enterococcus faecalis* strains and their characteristics.

Strain Code	Geographical area	Original Farm	Farm ID	Source of isolation	Sampling date	Host species	Health status	Antibiotic use recent (last 30 days before sample collection)	Housing	Sampling method
12EN	Kuyavian-Pomeranian	Farm A	KP_A	Raw milk	2022	Cow	Clinically healthy	No	Pasture	Sterile container
13EN	Kuyavian-Pomeranian	Farm B	KP_B	Raw milk	2022	Cow	Clinically healthy	No	Pasture	Sterile container
15EN	Kuyavian-Pomeranian	Farm A	KP_A	Raw milk	2022	Cow	Clinically healthy	No	Pasture	Sterile container
16EN	Kuyavian-Pomeranian	Farm C	KP_C	Raw milk	2022	Cow	Clinically healthy	No	Pasture	Sterile container
17EN	Kuyavian-Pomeranian	Farm C	KP_C	Raw milk	2022	Cow	Clinically healthy	No	Pasture	Sterile container
20EN	Kuyavian-Pomeranian	Farm H	KP_H	Raw milk	2022	Cow	Clinically healthy	No	Pasture	Sterile container
21EN	Kuyavian-Pomeranian	Farm A	KP_A	Raw milk	2022	Cow	Clinically healthy	No	Pasture	Sterile container
41EN	Kuyavian-Pomeranian	Farm A	KP_A	Raw milk	2022	Cow	Clinically healthy	No	Pasture	Sterile container
53EN	Kuyavian-Pomeranian	Farm A	KP_A	Raw milk	2022	Cow	Clinically healthy	No	Pasture	Sterile container
54EN	Warmian-Masurian	Farm A	WM_A	Raw milk	2022	Cow	Clinically healthy	No	Pasture	Sterile container
57EN	Kuyavian-Pomeranian	Farm C	KP_C	Raw milk	2022	Cow	Clinically healthy	No	Pasture	Sterile container
59EN	Warmian-Masurian	Farm B	WM_B	Raw milk	2022	Cow	Clinically healthy	No	Pasture	Sterile container
60EN	Kuyavian-Pomeranian	Farm D	KP_D	Raw milk	2022	Cow	Clinically healthy	No	Pasture	Sterile container
61EN	Kuyavian-Pomeranian	Farm D	KP_D	Raw milk	2022	Cow	Clinically healthy	No	Pasture	Sterile container
62EN	Kuyavian-Pomeranian	Farm D	KP_D	Raw milk	2022	Cow	Clinically healthy	No	Pasture	Sterile container
63EN	Kuyavian-Pomeranian	Farm E	KP_E	Raw milk	2022	Cow	Clinically healthy	No	Pasture	Sterile container
64EN	Warmian-Masurian	Farm C	WM_C	Raw milk	2022	Cow	Clinically healthy	No	Pasture	Sterile container
65EN	Warmian-Masurian	Farm D	WM_D	Raw milk	2022	Cow	Clinically healthy	No	Pasture	Sterile container
66EN	Kuyavian-Pomeranian	Farm F	KP_F	Raw milk	2022	Cow	Clinically healthy	No	Pasture	Sterile container
67EN	Kuyavian-Pomeranian	Farm H	KP_H	Raw milk	2022	Cow	Clinically healthy	No	Pasture	Sterile container
68EN	Warmian-Masurian	Farm H	WM_H	Raw milk	2022	Cow	Clinically healthy	No	Pasture	Sterile container
69EN	Warmian-Masurian	Farm C	WM_C	Raw milk	2022	Cow	Clinically healthy	No	Pasture	Sterile container
70EN	Kuyavian-Pomeranian	Farm I	KP_I	Raw milk	2022	Cow	Clinically healthy	No	Pasture	Sterile container
76EN	Kuyavian-Pomeranian	Farm G	KP_G	Raw milk	2022	Cow	Clinically healthy	No	Pasture	Sterile container
77EN	Kuyavian-Pomeranian	Farm G	KP_G	Raw milk	2022	Cow	Clinically healthy	No	Pasture	Sterile container
78EN	Warmian-Masurian	Farm K	WM_K	Raw milk	2022	Cow	Clinically healthy	No	Pasture	Sterile container
79EN	Kuyavian-Pomeranian	Farm F	KP_F	Raw milk	2022	Cow	Clinically healthy	No	Pasture	Sterile container
80EN	Kuyavian-Pomeranian	Farm F	KP_F	Raw milk	2022	Cow	Clinically healthy	No	Pasture	Sterile container
81EN	Kuyavian-Pomeranian	Farm F	KP_F	Raw milk	2022	Cow	Clinically healthy	No	Pasture	Sterile container
